# Antibiotic Resistance and Biofilm Production Capacity in *Clostridioides difficile*

**DOI:** 10.3389/fcimb.2021.683464

**Published:** 2021-08-04

**Authors:** Layan Abu Rahmoun, Maya Azrad, Avi Peretz

**Affiliations:** ^1^The Azrieli Faculty of Medicine, Bar Ilan University, Safed, Israel; ^2^Clinical Microbiology Laboratory, Baruch Padeh Medical Center, Poriya, Israel

**Keywords:** *C. difficile*, reduced antibiotic susceptibility, recurrence, metronidazole, vancomycin, biofilm production

## Abstract

**Background:**

*Clostridioides difficile* (*C. difficile*) is one of the primary pathogens responsible for infectious diarrhea. Antibiotic treatment failure, occurring in about 30% of patients, and elevated rates of antibiotic resistance pose a major challenge for therapy. Reinfection often occurs by isolates that produce biofilm, a protective barrier impermeable to antibiotics. We explored the association between antibiotic resistance (in planktonic form) and biofilm-production in 123 *C. difficile* clinical isolates.

**Results:**

Overall, 66 (53.6%) out of 123 isolates produced a biofilm, with most of them being either a strong (44%) or moderate (34.8%) biofilm producers. When compared to susceptible isolates, a statistically higher percentage of isolates with reduced susceptibility to metronidazole or vancomycin were biofilm producers (*p* < 0.0001, for both antibiotics). Biofilm production intensity was higher among tolerant isolates; 53.1% of the metronidazole-susceptible isolates were not able to produce biofilms, and only 12.5% were strong biofilm-producers. In contrast, 63% of the isolates with reduced susceptibility had a strong biofilm-production capability, while 22.2% were non-producers. Among the vancomycin-susceptible isolates, 51% were unable to produce biofilms, while all the isolates with reduced vancomycin susceptibility were biofilm-producers. Additionally, strong biofilm production capacity was more common among the isolates with reduced vancomycin susceptibility, compared to susceptible isolates (72.7% *vs.* 18.8%, respectively). The distribution of biofilm capacity groups was statistically different between different Sequence-types (ST) strains (*p* =0.001). For example, while most of ST2 (66.7%), ST13 (60%), ST42 (80%) isolates were non-producers, most (75%) ST6 isolates were moderate producers and most of ST104 (57.1%) were strong producers.

**Conclusions:**

Our results suggest an association between reduced antibiotic susceptibility and biofilm production capacity. This finding reinforces the importance of antibiotic susceptibility testing, mainly in recurrence infections that may be induced by a strain that is both antibiotic tolerant and biofilm producer. Better adjustment of treatment in such cases may reduce recurrences rates and complications. The link of biofilm production and ST should be further validated; if ST can indicate on isolate virulence, then in the future, when strain typing methods will be more available to laboratories, ST determination may aid in indecision between supportive *vs.* aggressive treatment.

## Introduction

*Clostridioides difficile* (*C. difficile*) is a Gram-positive, spore-forming, anaerobic bacteria, that constitutes one of the primary pathogens responsible for nosocomial diarrhea (Johnson and Gerding, 1998; De Roo and Regenbogen, 2020). *C. difficile* infection (CDI) is primarily induced by antibiotic therapy, which alters the gut microbiome (Khurana et al., 2020). Along with elevated CDI incidence, the evolution of CDI has changed over the past few decades, due to emergence of hypervirulent strains associated with increased disease severity and high complication and morbidity rates (Goudarzi et al., 2014). The major virulence factors contributing to CDI are toxin production and spore formation (Voth and Ballard, 2005; Rupnik et al., 2009; Semenyuk et al., 2014; Janoir, 2016). Ingestion of the highly resistant spores is the first step toward CDI onset, which is followed by spore germination in the gut and bacterial colonization (Semenyuk et al., 2014). Toxins A and B secreted by the vegetative cells, mediate CDI pathogenesis, which manifests as damage to intestinal cells and by a proinflammatory host response (Voth and Ballard, 2005; Goudarzi et al., 2014; Janoir, 2016).

The accepted treatments had long been vancomycin and metronidazole, with the last being the first line treatment (Vuotto et al., 2016). However, due to higher recurrences rates and lower clinical cure rates, compared to vancomycin, metronidazole was replaced in 2018 with oral vancomycin or fidaxomicin (McDonald et al., 2018). Paradoxically, antibiotics may induce further changes in the gut microbiome, resulting in recurrence of CDI, which occurs in approximately 25% of the patients following treatment completion (Johnson and Gerding, 1998; Johnson, 2009; Goudarzi et al., 2014). Patients with disease recurrence, are prone to episodic reappearances, with risks of 45% and 64% for second and third episodes, respectively (Surawicz and Alexander, 2011). Mostly, recurrence is associated with the original strain (Figueroa et al., 2012), assigned to either resistance to the administered antibiotic or to a protective, antibiotic-impermeable barrier it forms (Dawson et al., 2021). This barrier is composed of sessile, surface-associated microbial communities, known as a biofilm (Dawson et al., 2012). Previous studies have demonstrated *in vitro* and *in vivo* formation of biofilms by *C. difficile* (Lawley et al., 2009; Buckley et al., 2011; Dawson et al., 2012; Donelli et al., 2012; Dapa and Unnikrishnan, 2013; Ethapa et al., 2013; Semenyuk et al., 2014; Semenyuk et al., 2015; Crowther et al., 2016; Vuotto et al., 2016; Jain et al., 2017; Soavelomandroso et al., 2017; James et al., 2018; Poquet et al., 2018; Dawson et al., 2021; Normington et al., 2021), which have been shown to include polysaccharides, proteins and extracellular DNA (Dawson et al., 2021), as well as toxins and spores (Dapa and Unnikrishnan, 2013; Semenyuk et al., 2014) [Reviewed at (Frost et al., 2021)].

The biofilm has been suggested to play several roles, including evasion of the host immune system and protection from antimicrobials (Semenyuk et al., 2014; Hall and Mah, 2017). While various studies have reported on an association between biofilm and *C. difficile* antibiotic resistance in the biofilm form, there is scant evidence whether the antibiotic susceptibility of the planktonic cells is associated with their ability to produce a biofilm. This prompted us to investigate whether resistant isolates are more likely to form biofilms as compared to sensitive isolates.

## Materials and Methods

### Bacterial Isolates

This study was performed at the clinical microbiology laboratory of the Baruch Padeh Medical Center, Israel. One-hundred and twenty-three clinical *C. difficile* isolates were randomly chosen for this study. These isolates were previously isolated from stool samples of patients aged ≥18 years, who were diagnosed with CDI during hospitalization at the center, between January 2018 and April 2020. Poriya Baruch Padeh Medical Center Helsinki Committee, POR-0085-15, approved the study. A signed informed consent for participation was obtained from all patients.

Stool samples were inoculated on chromID™ *C. difficile* (bioMérieux, Durham, NC). Following incubation of the agar plates at 37°C under anaerobic conditions for 48 h, *C. difficile* colonies were identified using the Bruker Biotyper system (Bruker Daltonics, Bremen, Germany), which is based on the matrix-assisted laser desorption ionization-time of flight (MALDI TOF) technique (Singhal et al., 2015).

### Antibiotic Susceptibility Testing

Antimicrobial susceptibility testing (AST) was performed using the Etest technique, which determines the minimum inhibitory concentration (MIC), i.e., the minimal antibiotic concentration that inhibits the growth of bacteria under specific conditions. To this end, *C. difficile* colonies were suspended in Thioglycolate broth medium (Becton Dickinson, Heidelberg, Germany) until 1.0 McFarland turbidity. The inoculum was then seeded on Brucella blood agar growth medium (Hy Laboratories, Rehovot, Israel). Gradient Etest strips (bioMérieux, Durham, NC) of vancomycin and metronidazole were added to the plates. Following incubation under anaerobic conditions, at 37°C, for 24 h, the MIC was determined for each antibiotic. Test results (susceptible/with reduced susceptibility) were interpreted according to the European Committee on Antimicrobial Susceptibility Testing (EUCAST) recommendations (EUCAST, 2021), that determined the following epidemiological cut-offs- reduced susceptibility for vancomycin is defined with an MIC above 2 µg/mL; reduced susceptibility to metronidazole is defined with an MIC above 2 µg/mL.

**Table S1** presents all the collected data regarding the study’s isolates (antibiotic susceptibility, ST strain and biofilm production capacity). **Table S2** presents a comparison of MIC between Etest and micro broth dilution, for the validation of Etest.

### Microtiter Plate Assay for the Assessment of Biofilm Production

Biofilm production by the different strains was assessed using a microtiter plate, as previously described (Hammond et al., 2014). Each *C. difficile* strain was cultured in brain heart infusion agar supplemented with yeast extract + 0.1% L-cysteine (BHIS), at 37°C, for 24 h. A sample was taken from the BHIS broth and normalized to an OD_600nm_ of 0.8. Then, the inoculum was diluted 1:100 in BHIS broth and a 200 μl aliquot of each diluted inoculum was dispensed into a 96-well Nunc flat-bottom microtiter plate.

BHIS was used as a negative control and the biofilm-forming ATCC^®^ BAA-1382 *C. difficile* (strain 630) was used as a positive control. The plate was incubated at 37°C, for 24 h, in an anaerobic cabinet. Following incubation, spent medium was removed and wells were washed twice with phosphate buffered saline (PBS) (Oxoid, Cambridge, UK). PBS was discarded and 200 μl of 0.25% (w/v) aqueous crystal violet were added to each well. After 5 min, the wells were washed with PBS eight times and air-dried. Then, 200 μl ethanol: acetone (1:1) solution was added to each well in order to solubilize the dye from adherent cells. Absorbance was measured within 5 min, at 570 nm, using an ELISA reader (Multiskan Go, Fisher-Scientific Ltd., Vantaa, Finland). The cut-off OD_570_ (ODc) was determined as three standard deviations above the mean OD of the negative control.

The isolates were classified as: non-producers - isolates with OD<OD_C_, weak producers - isolates with ODc < OD ≤ 2× ODc, moderate producers - 2× ODc < OD ≤ 4× ODc and strong producers - OD> 4× ODc (Stepanovic et al., 2000). According to this calculation, the following ranges of OD_570_ were used: Non producers- OD < 0.35, Weak producers- 0.36< OD< 0.7, Moderate producers- 0.71< OD< 1.4, Strong producers- OD> 1.41. The OD_570_ range of the positive control strain 630 was 2.1-2.4.

The experiment was repeated 3 times, with triplicates of each strain tested in each experiment.

### Multi-Locus Sequence Typing (MLST) of Bacterial Isolates

Total genomic DNA was extracted from bacterial isolates using the QIAamp DNA Mini Kit (QIAGEN, Hilden, Germany), according to the manufacturer’s instructions. MLST was performed as previously described (Griffiths et al., 2010):

A. Amplification: The DNA of 7 housekeeping genes of each isolate was amplified in a polymerase chain reaction (PCR) using the qPCRBIO SyGreen Blue Mix Hi-ROX kit (PCR Biosystems Inc., Wayne, Pennsylvania, USA), on a real-time PCR device (BioRad CFX96 Real-Time Detection System, Hercules, CA, USA). Amplification conditions were: 95°C for 2 min, followed by 40 cycles of (95°C for 5 s, and 60–65°C for 20–30 s).

B. Sequencing and Sequence Type (ST) Determination: the 7 PCR amplicons were purified and their nucleotide sequences were determined using amplification primers, as previously described (Griffiths et al., 2010). The capillary electrophoresis method was used for genotyping on an ABI PRISM^®^ 310 Genetic Analyzer (Applied Biosystems, Foster City, CA, USA). Data analysis was performed using ChromasLite v2.01 (Technelysium DNA Sequencing Software, South Brisbane, Australia) and Sequencher v5.1 (Gene Codes Corporation, Ann Arbor, USA).

Following sequencing, the allelic numbers of each gene and the STs were determined using the PubMLST *C. difficile* database (http://pubmlst.org/cdifficile/). Each ST number was determined for each specific combination of alleles.

### Statistical Analysis

Chi-square test was applied for analyzing differences in the proportions of the different categories of biofilm production capacity, between susceptible isolates and isolates with reduced susceptibility to metronidazole and vancomycin. **Tables S3** and **S4** presents the expected and observed values for each Chi square analysis.

Fisher’s Exact Test was performed to determine if there were ST specific differences in the biofilm production capacity (*p*<0.05 indicates there were ST specific differences). Fisher’s exact test was performed to determine if there were ST specific differences in antibiotic susceptibility (*p*<0.05 indicates there were ST specific differences).

The Analysis of Variance (ANOVA) test was applied for analyzing differences in MIC values between the different isolates’ groups according to the biofilm producing capacity.

All assays were repeated 3 times (n=3). All tests applied were two-tailed, and a *p* value of 5% or less was considered statistically significant. The data was analyzed using the SAS^®^ version 9.3 (SAS Institute, Cary, North Carolina). Additional data can be found in the **Supplementary File**.

## Results

### Sequence Types of Study’s Isolates

Following MLST analysis, we categorized the isolates into eight major ST groups (each group containing at least 5 isolates) and additional group called “others”, which combined several different STs (with less than 5 isolates per ST). The most common ST among our isolates was ST4 (24/119, 20.2%), followed by ST42 (10/119, 8.4%), ST13 (10/119, 8.4%), ST37 (9/119, 7.6%), ST6 (8/119, 6.7%), ST1 (7/119, 5.9%), ST104 (7/119, 5.9%) and ST2 (6/119, 5%).

### Antibiotic Susceptibility Profiles

Of the 123 tested clinical *C. difficile* isolates, 27 (22%) showed reduced susceptibility to metronidazole (**Table 1**). Average and median MIC of the metronidazole-susceptible isolates were 0.4 μg/mL and 0.25 μg/mL, respectively. In contrast, isolates with reduced susceptibility to metronidazole had an average MIC of 246.7 μg/mL and a median MIC of 256 μg/mL.

**Table 1:** Antibiotic susceptibility profiles of study isolates.

Antibiotic/Characteristics	Metronidazole		Vancomycin	
	Susceptible Isolates (MIC≤ 2) n=96	Isolates with Reduced Susceptibility (MIC>2) n=27	Susceptible Isolates (MIC≤ 2) n=112	Isolates with Reduced Susceptibility (MIC>2) n=11
Average MIC, (μg/mL)	0.4	246.7	0.6	118.7
Median MIC (Q1, Q4^*^), (μg/mL)	0.25 (0.125, 1.5)	256 (256, 256)	0.38 (0.25, 2)	8 (4, 256)

^*^Q1, Q4 indicate quartiles 1 and 4, respectively.

Reduced susceptibility to vancomycin was found in 11 (9%) isolates. Average and median MIC of the vancomycin-susceptible isolates were 0.6 μg/mL and 0.38 μg/mL, respectively. The isolates with reduced susceptibility to vancomycin had an average and a median MIC of 118.7 μg/mL and 8 μg/mL, respectively (**Table 1**). **Figures S1** and **S2** present the distribution of metronidazole and vancomycin MICs for all isolates, respectively. MIC of the control strain 630 was 0.094 μg/mL and 1 μg/mL for metronidazole and vancomycin, respectively.

Next, we tested whether there were ST specific differences in antibiotic susceptibility. We found that metronidazole susceptibility pattern was diverse between different ST (*p*= 0.015) (**Table S5**). Specifically, ST104 accounted for most of the differences. For vancomycin, the differences in susceptibility pattern between STs were not statistically significant.

### Biofilm Production Capacity

Overall, 53.6% (66/123) isolates were able to produce a biofilm (**Table 2**). Most of the biofilm-producing isolates were either strong (29/66, 44%) or moderate (23/66, 34.8%) biofilm producers.

**Table 2:** Biofilm production capacity of study isolates.

	Non-biofilm producing isolates	Biofilm-producing isolates		
		Weak producers	Moderate producers	Strong producers
**N (%)**	57 (46.3)	14 (11.4)	23 (18.7)	29 (23.6)

### Association Between ST and Biofilm Production Capacity

We compared the biofilm production capacity of isolates from different STs and found that the distribution of biofilm capacity groups (non-producers, weak-, moderate- and strong producers) was statistically different between the different STs (*p* =0.001) (**Table 3**). For example, while most isolates of ST13 (60%), ST42 (80%) and ST2 (66.7%) were non-producers, most (75%) isolates of ST6 were moderate producers and most of ST104 (57.1%) isolates were strong producers. One isolate which belong to ST43 (mean OD_570 =_ 2.22) produced a biofilm as strong as the control 630 strain which belong to ST54 (Saito et al., 2019). Two other isolates that belong to ST8 and ST239 had a stronger biofilm production capacity, compared to the control strain (mean OD_570 =_ 3.93 and 3.34, respectively). All three strains, as well as the control strain 630, belong to clade 1.

**Table 3:** Biofilm production capacity among the different STs.

ST*(%)	Non-biofilm producing isolates N(%)	Biofilm-producing isolates			Total N (%)	*p* value**
		Weak producers N(%)	Moderate producers N(%)	Strong producers N(%)		
1 (5.9)	1 (14.3)	3 (42.9)	3 (42.9)	0	7 (5.9)	0.001
2 (5)	4 (66.7)	1 (16.7)	0	1 (16.7)	6 (5)	
4 (20.2)	13 (54.2)	2 (8.3)	2 (8.3)	7 (29.2)	24 (20.2)	
6 (6.7)	2 (25)	0	6 (75)	0	8 (6.7)	
13 (8.4)	6 (60)	1 (10)	3 (30)	0	10 (8.4)	
37 (7.6)	4 (44.4)	1 (11.1)	0	4 (44.4)	9 (7.6)	
42 (8.4)	8 (80)	0	0	2 (20)	10 (8.4)	
104 (5.9)	3 (42.9)	0	0	4 (57.1)	7 (5.9)	
Others^***^ (31.9)	15 (39.5)	3 (7.9)	9 (23.7)	11 (28.9)	38 (31.9)	

^*^The MLST analysis of 4 isolates is missing.

^**^ Fisher’s Exact Test was performed to determine if there were ST specific differences in the biofilm production capacity; p value < 0.05.

*** Others= ST3, ST8, ST10, ST12, ST17, ST35, ST43, ST54, ST55, ST59, ST60, ST103, ST153, ST239, ST421 and ST439.

### Association Between Antibiotic Resistance and Biofilm Production Capacity

**Figure 1:**
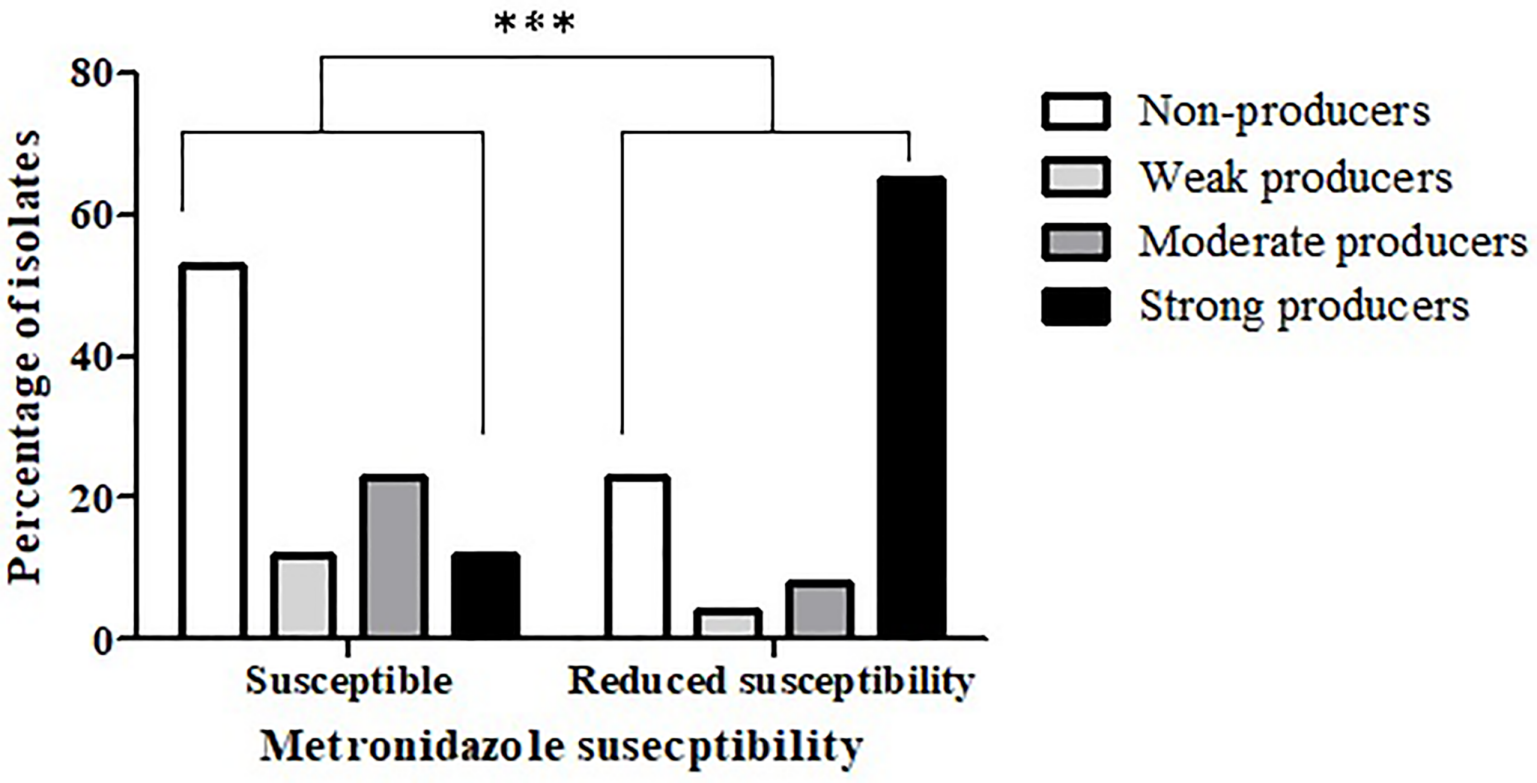
Biofilm production capacity among metronidazole-susceptible isolates and isolates with reduced susceptibility. The biofilm production capacity of *C. difficile* isolates was compared between metronidazole-susceptible (n = 96) and reduced metronidazole susceptibility isolates (n = 27) (***p < 0.0001). The *p* value represents the comparison of the biofilm production capacity between susceptible isolates and isolates with reduced susceptibility (Chi square analysis).

Comparison of biofilm production capacity between isolates that were metronidazole-susceptible *versus* isolates with reduced susceptibility to metronidazole, revealed a significantly higher rate of biofilm producers among isolates with reduced susceptibility (*p* < 0.0001) (**Figure 1**). Specifically, 53.1% (51/96) of the metronidazole-susceptible isolates were not able to produce biofilms, and only 12.5% (12/96) were strong biofilm-producers. In contrast, most (63%, 17/27) of the isolates with reduced susceptibility had a strong biofilm-producing capacity, while 22.2% (6/27) isolates were non-producers.

**Figure 2:**
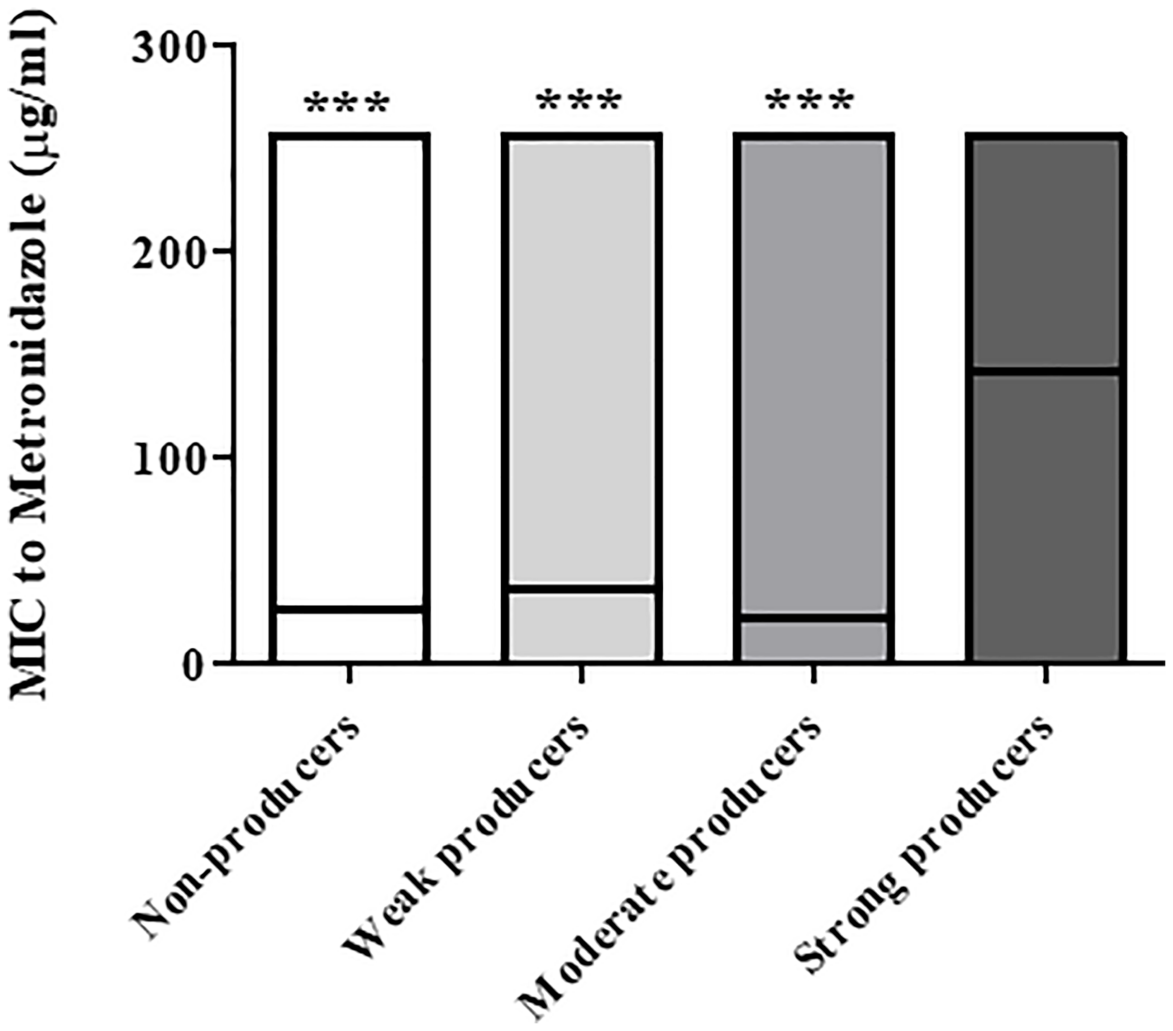
Distribution of Metronidazole MIC values of non-biofilm producers, weak-, moderate- and strong biofilm-producing *C. difficile* isolates. The minimal, maximal and average MIC values are shown (the average is indicated by the line within the bar) for each group of isolates (non-producers, weak-, moderate- and strong producers’ groups). The mean metronidazole MIC of strong biofilm-producing isolates (n = 29) were significantly higher compared to MIC values of isolates that were non-producers (n = 57), weak producers (n = 14) and moderate producers (n = 23), ***p < 0.001. The *p* value represents the comparison of the mean MIC between the four categories of biofilm production capacity (ANOVA analysis).

**Figure 2** presents the distribution of metronidazole MIC values among the different isolates in relation to their biofilm production capacity. Strong biofilm producers had statistically higher mean MIC (141.6 μg/mL) compared to all other biofilm production categories (27.3, 36.8, and 22.6 μg/mL for non-producers, weak producers and moderate producers, respectively; *p* < 0.0001).

**Figure 3:**
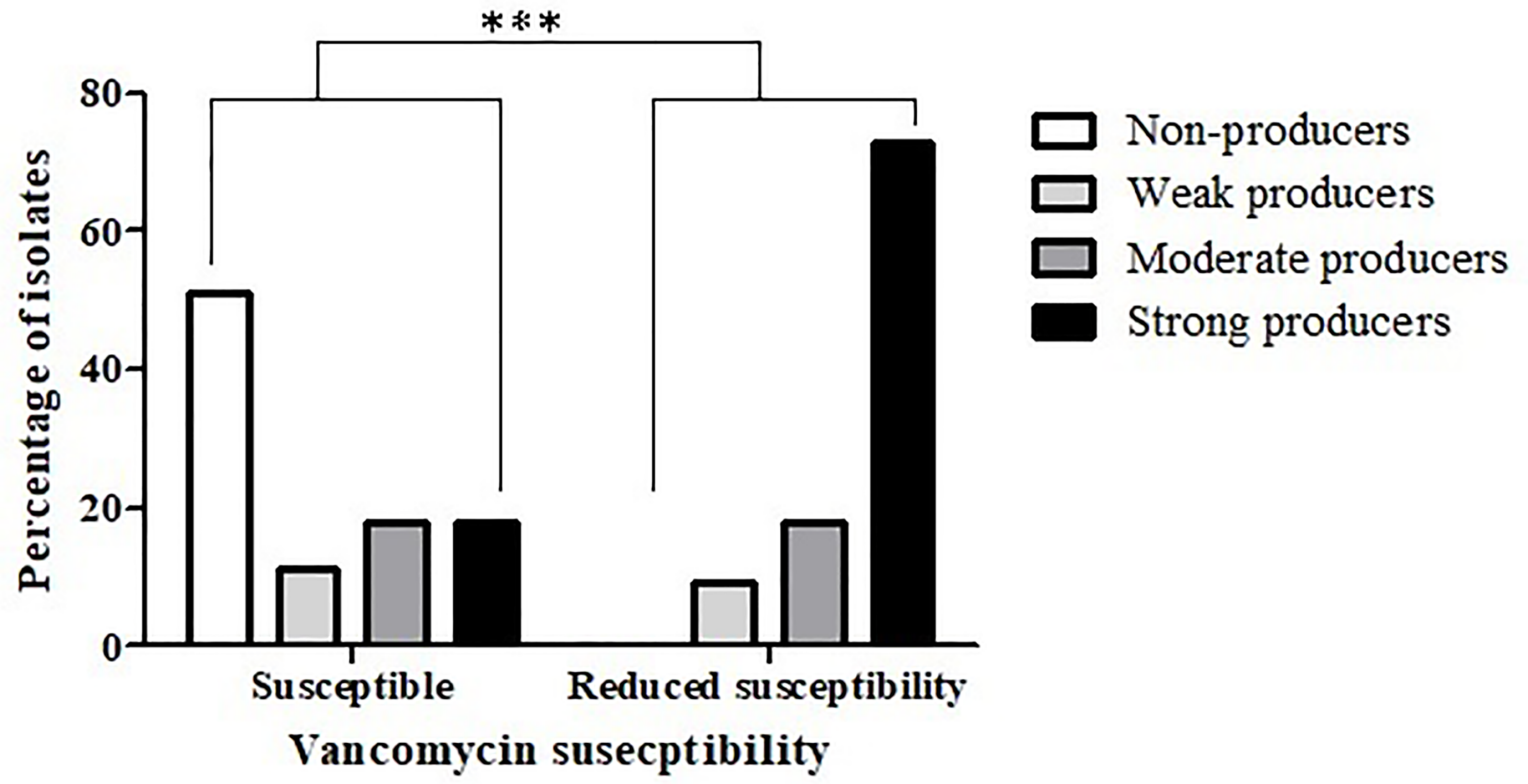
Biofilm production capacity among isolates that are vancomycin-susceptible and isolates with reduced vancomycin- susceptibility. The biofilm-production capacity of *C. difficile* isolates was compared between isolates that are vancomycin-susceptible (n = 112) and isolates with reduced vancomycin susceptibility (n = 11) isolates (***p < 0.0001). The p value represents the comparison of the biofilm production capacity between susceptible isolates and isolates with reduced susceptibility (Chi square analysis).

An association between reduced antibiotic susceptibility and biofilm production capacity was also noted in relation to vancomycin (*p* < 0.0004) (**Figure 3**); while 51% (57/112) of the vancomycin-susceptible isolates were not able to produce biofilms, all the isolates with reduced vancomycin susceptibility were biofilm-producers. Additionally, strong biofilm production capacity was more common among the isolates with reduced vancomycin susceptibility, compared to the susceptible isolates (72.7% *vs.* 18.8%, respectively).

**Figure 4:**
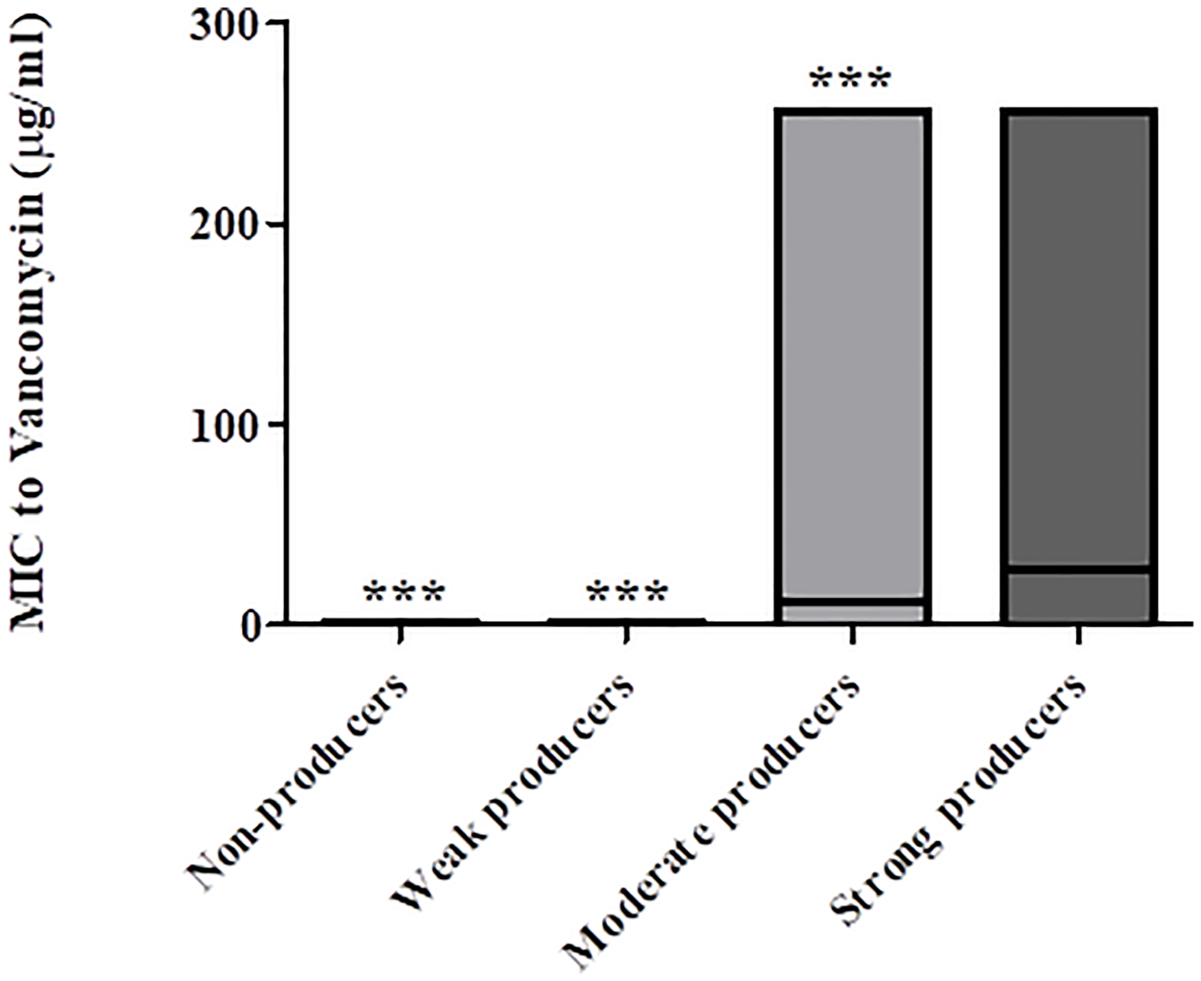
Distribution of vancomycin MIC values of non-biofilm producers, weak-, moderate- and strong biofilm producers. The minimal, maximal and average MIC values are shown (the average is indicated by the line within the bar) for each group of isolates (non-producers, weak-, moderate- and strong producers’ groups). The mean vancomycin MIC of strong biofilm producers (n = 29) were significantly higher as compared to MIC values of isolates that were non-producers (n = 57), weak producers (n = 14) and moderate producers (n = 23), ***p < 0.001. The *p* value represents the comparison of the mean MIC between the four categories of biofilm production capacity (ANOVA analysis).

The distribution of vancomycin MIC values also indicated an association between reduced vancomycin susceptibility and biofilm-producing capacity (**Figure 4**); strong biofilm-producing isolates had higher mean vancomycin MIC (28.9 μg/mL) than non-producers, weak- and moderate- biofilm producers (0.5 μg/mL, 20.3 μg/mL, and 12.2 μg/mL, respectively; *p* < 0.0001).

## Discussion

*C. difficile* biofilm production has been associated with disease recurrence, which poses a major challenge for CDI treatment (Dapa and Unnikrishnan, 2013). Although *C. difficile* biofilms have been studied both *in vitro* and *in vivo*, most of them explored only a limited number of strains (Lawley et al., 2009; Buckley et al., 2011; Dawson et al., 2012; Dapa and Unnikrishnan, 2013; Ethapa et al., 2013; Semenyuk et al., 2014; Semenyuk et al., 2015; Crowther et al., 2016; Vuotto et al., 2016; Soavelomandroso et al., 2017; James et al., 2018; Poquet et al., 2018; Dubois et al., 2019; Dawson et al., 2021; Normington et al., 2021). Additionally, previous studies have not compared the biofilm production capacity of antibiotic-susceptible versus antibiotic-tolerant isolates.

In the current study, we reported on the antibiotic susceptibility and biofilm-production capacity of 123 C*. difficile* isolates. This information is novel, considering the fact that most *in vitro* studies of *C. difficile* biofilms focused on 3-7 different well-characterized strains (Dapa and Unnikrishnan, 2013; Ethapa et al., 2013; Semenyuk et al., 2014; Vuotto et al., 2016; James et al., 2018). We found that 53.6% of the tested isolates were able to produce a biofilm. This finding is interesting in light of the fact that only one previous study has found non-biofilm producers among *C. difficile* isolates and their percentage was very low (2/102, 1.9%) (Tijerina-Rodriguez et al., 2019). However, 81.4% of the isolates in this study belonged to ribotype 027 (RT027, ST1), which is known to produce biofilm (Semenyuk et al., 2014). Another previously reported study that profiled 37 isolates, applied different biofilm production classification system, and had no category of non-producers (Pantaleon et al., 2018). Regarding the other categories of biofilm production, Tijerina-Rodriguez et al., 2019 found that 87.25% of the study’s isolates were strong producers (Tijerina-Rodriguez et al., 2019). As mentioned earlier, most of these isolates were RT027. Thus, is it possible that these isolates originated from the same strain. In the study of Pantaleon et al., 2018, the distribution of biofilm production capacity was: 50%- low, 17.6%-moderate and 32.4%-strong capacity (Pantaleon et al., 2018). Although the distribution differs from our results, it is difficult to compare the isolates’ biofilm production capacity, since we used different classification system.

Interestingly, we found an association between the biofilm capacity to the ST. However, this association should further be explored due to the low number of isolates in each ST group. A former study with 37 isolates, has not found an association between biofilm production and ST, notwithstanding biofilm production capacity was variable among the different strains (Pantaleon et al., 2018). In the same study, strong biofilm- producing isolates belonged to ST3 (RT027), ST19 (RT05), ST21 (RT03), ST26 (RT156), ST33 (RT095), ST34 (RT014/020) and ST36 (RT165) (Pantaleon et al., 2018). In contrast, our strong biofilm- producing isolates belonged to other STs including ST2, ST4, ST8, ST37, ST42, ST43, ST55, ST59, ST60, ST104, ST239, ST421, and ST439. In another study, RT014 (ST 49/13/2) and RT017 (ST37/45) were found to be strong biofilm producers (Kullin et al., 2016). It is important to note that most of these ST are genetically different and even strains that belong to the same phylogenetic clade are genetically divergent due to frequent horizontal gene transfer and homologous recombination (Didelot et al., 2012; Stabler et al., 2012). On the other hand, we noticed that most strong producers in our study and in a previous study (Pantaleon et al., 2018) belong to clade 1. In another study which investigated biofilm production and structure in five strains representative of the five phylogenetic lineages, the strongest biofilm producer, strain RT012 (our control strain 630), also belong to clade 1 (Dawson et al., 2021). Similar biofilm formation capabilities were seen in RT023 and RT078, that belong to clade 3 and to the most divergent clade 5, respectively (Dawson et al., 2021). In the current study, ST37 which belong to clade 4 also presented high biofilm production capacity. Thus, further studies should be performed in order to confirm whether there is an association between ST and biofilm production or alternatively between clade and biofilm production. If such an association truly exists, ST determination at the beginning of CDI may aid in identification of hypervirulent isolates and rapid adjustment of specific treatment.

The main aim of the current work was to test whether isolates with reduced susceptibility to antibiotics have a greater biofilm-producing capacity than susceptible isolates. We found a higher percentage of biofilm producers among isolates with reduced metronidazole- or vancomycin- susceptibility as compared to the susceptible isolates. Furthermore, isolates with reduced metronidazole or vancomycin isolates were characterized with a stronger biofilm-producing capacity as compared to susceptible isolates. To the best of our knowledge, this is the first time that biofilm production capacity is associated with reduced antibiotic susceptibility in *C*. *difficile* planktonic cells. Most studies have determined the antibiotic susceptibility of *C. difficile* biofilms, showing that biofilm cells are more tolerant of several antibiotics, compared to their planktonic counterparts (Dawson et al., 2012; Dapa and Unnikrishnan, 2013; Crowther et al., 2014; Semenyuk et al., 2014; Dawson et al., 2021). In contrast, we defined the antibiotic susceptibility of the different isolates before biofilm production, and associated the susceptibility profile with the biofilm-forming capacity. Similar findings were reported in other bacterial species (Arciola et al., 2005; Klingenberg et al., 2005; de Araujo et al., 2006; Agarwal and Jain, 2012; Yekani et al., 2017; Ratajczak et al., 2021). For example, *Staphylococcus epidermidis* strains that produced exopolysaccharide had a significantly higher prevalence of resistance to aminoglycosides, sulfamethoxazole and ciprofloxacin, compared to non-producing isolates. Additionally, multiple resistance to antibiotics was more common among biofilm-producing isolates (Arciola et al., 2005). In a previous study with *Pseudomonas aeruginosa*, biofilm-producing isolates showed higher resistance rate to β-lactams and aminoglycosides antibiotics than non-biofilm-producing isolates. Additionally, biofilm production capacity and multi drug resistance (MDR) were significantly associated (Yekani et al., 2017).

Our findings arouse a question whether reduced antibiotic susceptibility and biofilm production are regulated by the same pathways. Alternatively, it is possible that genes conferring reduced antibiotic susceptibility and genes that are associated with biofilm production pathway are co-located on the same genetic element. Although there are no supportive evidences in *C. difficle* to our hypothesis, several reports in other species may reinforce our assumptions; for example, genes encoding the flagella and fimbriae proteins in *Escherichia coli* (*E. coli*) O157:H7 were found to be located on plasmids (Karch et al., 1987). Since some antibiotic resistance genes are also located in plasmids, it is possibly that the same plasmid constitute genes of both traits. Another possible explanation which also concern with plasmids is the promotion of biofilm formation by conjugative plasmids [reviewed at (Madsen et al., 2012)]; it was shown that factors expressed by conjugative plasmid induced biofilm in *E. coli* and removal of this plasmid resulted in inability of biofilm formation (Ghigo, 2001). These results were later supported by another study in which co-culture of plasmid-bearing and non-bearing *E. coli* strains resulted in gaining of the biofilm production capacity among the non-bearing isolates. The authors suggested that this capacity was acquired by conjugative plasmid transfer (Reisner et al., 2006).

Interestingly, biofilm development was shown to be influenced by efflux pumps presence. A previous study in *Salmonella enterica* isolates induced mutations in the efflux pumps that contribute to tetracycline resistance. As a result of the pumps’ loss or inhibition, mutants were unable to produce curli, one of the biofilm’s component in *Salmonella*. Further experiments revealed that mutants had a transcriptional repression of the two curli biosynthetic operons. The authors proposed there was a link between the regulation of multidrug efflux and biofilm formation *via* global regulators of efflux (Baugh et al., 2012; Baugh et al., 2014).

Since mechanisms of *C. difficile* antibiotic resistance to both metronidazole and vancomycin are not clearly understood, it is possible that efflux pumps or plasmids that harbor antibiotic resistance genes are part of these mechanisms. As mentioned above, the presence of either efflux pumps or plasmids may contribute to biofilm production. Therefore, the association of antibiotic resistance and biofilm production in *C. difficle* should be further investigated in order to find the exact mechanism that is responsible for this association. Nevertheless, this association between reduced susceptibility to antibiotics and biofilm production reinforces the importance of performing antibiotic susceptibility testing. This is mainly necessary in recurrence infections that may be induced by a strain that has both reduced susceptibilities to antibiotics and biofilm production capacity, thus has multiple pathogenicity traits. Better adjustment of antimicrobial treatment in such cases may reduce recurrences rates and complications.

It is known that biofilm production, as well as alterations in antibiotic targets and/or in metabolic routes/paths that directly confer antibiotic resistance can be induced by *in vivo* exposure to antibiotics (Peng et al., 2017). Sub-inhibitory concentrations of metronidazole (Semenyuk et al., 2014; Vuotto et al., 2016) and vancomycin (Ethapa et al., 2013) were shown to induce biofilm production in several *C. difficile* strains. Thus, a future study should investigate whether isolates that presented reduced susceptibility to metronidazole/vancomycin and biofilm-producing capacity, were isolated from patients that were previously treated with these antibiotics. Additionally, mutations in genes that confer resistance to antibiotics should be investigated.

The study has some limitations; first, it should be noted that a previous study reported on a heme-dependent increase in the metronidazole-MIC of *C. difficile* isolates for metronidazole, which in some cases led to interpretation of reduced susceptibility (MIC > 2 mg/L) (Boekhoud et al., 2020). Therefore, it is necessary to validate the MIC values of all the isolates with metronidazole- reduced susceptibility. Second, we have not performed sequencing thus we do not have information regarding the presence of specific resistance genes or plasmids that are known to be associated with antibiotic resistance.

In summary, this study correlated between antibiotic susceptibility and biofilm production, two main pathogenicity traits of *C. difficile*. Additionally, we found a link between ST to antibiotic susceptibility and to biofilm production. Further studies should be performed to confirm these finding and to reveal the mechanisms responsible for these associations. If ST can indicate on isolate virulence, then in the future, when strain typing methods will be more available to laboratories, ST determination may aid in indecision between supportive *vs.* aggressive treatment.

## Data Availability Statement

The original contributions presented in the study are included in the article/**Supplementary Material**. Further inquiries can be directed to the corresponding author.

## Ethics Statement

The studies involving human participants were reviewed and approved by Poriya Baruch Padeh Medical Center Helsinki Committee, POR-0085-15. The patients/participants provided their written informed consent to participate in this study.

## Author Contributions

Conceptualization, LA, MA, and AP. Data curation, LA and MA. Formal analysis, LA and MA. Methodology, LA, MA, and AP. Project administration, MA, and AP. Supervision, MA, and AP. Validation, LA, and MA. Visualization, LA, MA, and AP. Writing—original draft preparation, LA, MA, and AP. Writing—review and editing, LA, MA, and AP. All authors contributed to the article and approved the submitted version.

## Conflict of Interest

The authors declare that the research was conducted in the absence of any commercial or financial relationships that could be construed as a potential conflict of interest.

## Publisher’s Note

All claims expressed in this article are solely those of the authors and do not necessarily represent those of their affiliated organizations, or those of the publisher, the editors and the reviewers. Any product that may be evaluated in this article, or claim that may be made by its manufacturer, is not guaranteed or endorsed by the publisher.
