# Supplementary material for: Antibiotic Resistance and Biofilm Production Capacity in Clostridioides difficile
Source: Front Cell Infect Microbiol. 2021 Aug 4;11:683464. doi: 10.3389/fcimb.2021.683464 (PMC8371447; doi:10.3389/fcimb.2021.683464)
Supplement: Supplementary file 1 [file DataSheet_1.docx]

Reduced

susceptibility

**Figure S1: Metronidazole MIC distribution of study isolates**

**Figure S2: Vancomycin MIC distribution of study isolates**

**Table S1: ST, antibiotic susceptibility and biofilm production capacity of study's isolates**

| Isolate No. | ST | Clade | Metronidazole  MIC- µg/ml | Metronidazole  S\R^*^ | Vancomycin  MIC- µg/ml | Vancomycin  S\R | Biofilm production capacity^&^ |
| --- | --- | --- | --- | --- | --- | --- | --- |
| 1 | 42 | 1 | 0.5 | S | 0.5 | S | 3 |
| 2 | 43 | 1 | 0.38 | S | 0.38 | S | 0 |
| 3 | 37 | 4 | 0.75 | S | 0.38 | S | 0 |
| 4 | 10 | 1 | 0.25 | S | 0.25 | S | 0 |
| 5 | 43 | 1 | 256 | R | 0.75 | S | 0 |
| 6 | ND^#^ |  | 0.047 | S | 0.25 | S | 1 |
| 7 | 4 | 1 | 0.5 | S | 1 | S | 2 |
| 8 | 4 | 1 | 0.75 | S | 1.5 | S | 0 |
| 9 | 42 | 1 | 0.25 | S | 0.25 | S | 0 |
| 10 | 4 | 1 | 256 | R | 1 | S | 0 |
| 11 | 104 | 1 | 0.25 | S | 0.5 | S | 0 |
| 12 | 2 | 1 | 256 | R | 0.5 | S | 3 |
| 13 | 54 | 1 | 0.75 | S | 0.5 | S | 0 |
| 14 | 4 | 1 | 0.75 | S | 1 | S | 0 |
| 15 | 17 | 1 | 0.125 | S | 0.25 | S | 2 |
| 16 | 4 | 1 | 0.5 | S | 0.75 | S | 3 |
| 17 | 4 | 1 | 0.75 | S | 1.5 | S | 0 |
| 18 | 4 | 1 | 1 | S | 0.5 | S | 0 |
| 19 | 3 | 1 | 0.38 | S | 0.5 | S | 3 |
| 20 | 4 | 1 | 1 | S | 0.75 | S | 0 |
| 21 | 4 | 1 | 0.75 | S | 0.75 | S | 0 |
| 22 | 103 | 1 | 1 | S | 0.38 | S | 2 |
| 23 | 4 | 1 | 1.5 | S | 1 | S | 0 |
| 24 | 13 | 1 | 0.064 | S | 0.25 | S | 2 |
| 25 | 42 | 1 | 0.38 | S | 0.75 | S | 0 |
| 26 | 4 | 1 | 0.064 | S | 0.38 | S | 1 |
| 27 | 4 | 1 | 0.75 | S | 1 | S | 3 |
| 28 | 54 | 1 | 0.094 | S | 0.38 | S | 0 |
| 29 | 37 | 4 | 256 | R | 0.75 | S | 3 |
| 30 | 104 | 1 | 0.38 | S | 0.38 | S | 0 |
| 31 | 4 | 1 | 0.75 | S | 0.75 | S | 2 |
| 32 | 421 | 1 | 0.5 | S | 0.19 | S | 0 |
| 33 | 55 | 1 | 0.38 | S | 0.25 | S | 3 |
| 34 | 421 | 1 | 0.5 | S | 0.38 | S | 0 |
| 35 | 2 | 1 | 0.38 | S | 0.38 | S | 0 |
| Isolate No. | **ST** | **Clade** | **Metronidazole**  **MIC- µg/ml** | **Metronidazole**  **S\R** | **Vancomycin**  **MIC- µg/ml** | **Vancomycin**  **S\R** | **Biofilm production capacity** |
| 36 | 6 | 1 | 256 | R | 0.5 | S | 0 |
| 37 | 2 | 1 | 0.125 | S | 0.38 | S | 1 |
| 38 | 37 | 4 | 0.75 | S | 0.016 | S | 0 |
| 39 | 2 | 1 | 0.094 | S | 0.25 | S | 0 |
| 40 | 439 | 1 | 0.75 | S | 1 | S | 0 |
| 41 | 1 | 2 | 256 | R | 1 | S | 0 |
| 42 | 4 | 1 | 256 | R | 1 | S | 0 |
| 43 | 1 | 2 | 0.75 | S | 1 | S | 2 |
| 44 | 8 | 1 | 0.19 | S | 0.19 | S | 2 |
| 45 | 37 | 4 | 256 | R | 256 | R | 1 |
| 46 | 37 | 4 | 0.75 | S | 0.25 | S | 3 |
| 47 | 1 | 2 | 256 | R | 1.5 | S | 1 |
| 48 | 37 | 4 | 0.19 | S | 0.016 | S | 0 |
| 49 | 6 | 1 | 0.123 | S | 0.38 | S | 2 |
| 50 | 42 | 1 | 0.094 | S | 0.25 | S | 0 |
| 51 | 239 | 1 | 0.19 | S | 0.38 | S | 3 |
| 52 | 42 | 1 | 256 | R | 256 | R | 3 |
| 53 | ND |  | 0.032 | S | 0.25 | S | 1 |
| 54 | 104 | 1 | 256 | R | 1.5 | S | 0 |
| 55 | ND |  | 0.125 | S | 0.19 | S | 0 |
| 56 | 35 | 1 | 0.125 | S | 0.19 | S | 1 |
| 57 | 13 | 1 | 0.125 | S | 0.38 | S | 0 |
| 58 | 13 | 1 | 0.032 | S | 0.25 | S | 0 |
| 59 | 13 | 1 | 0.125 | S | 0.38 | S | 0 |
| 60 | 8 | 1 | 256 | R | 1.5 | S | 3 |
| 61 | 439 | 1 | 256 | R | 256 | R | 3 |
| 62 | 421 | 1 | 0.094 | S | 0.38 | S | 0 |
| 63 | 104 | 1 | 256 | R | 1.5 | S | 3 |
| 64 | ND |  | 0.125 | S | 1.5 | S | 1 |
| 65 | 17 | 1 | 256 | S | 256 | R | 2 |
| 66 | 3 | 1 | 1.5 | S | 0.38 | S | 1 |
| 67 | 37 | 4 | 4 | R | 0.19 | S | 3 |
| 68 | 12 | 1 | 0.5 | S | 0.25 | S | 0 |
| 69 | 55 | 1 | 0.38 | S | 0.38 | S | 0 |
| 70 | 6 | 1 | 0.125 | S | 0.5 | S | 2 |
| 71 | 42 | 1 | 0.25 | S | 0.25 | S | 0 |
| 72 | 55 | 1 | 0.094 | S | 0.19 | S | 0 |
| Isolate No. | **ST** | **Clade** | **Metronidazole**  **MIC- µg/ml** | **Metronidazole**  **S\R** | **Vancomycin**  **MIC- µg/ml** | **Vancomycin**  **S\R** | **Biofilm production capacity** |
| 73 | 35 | 1 | 0.75 | S | 0.25 | S | 0 |
| 74 | 4 | 1 | 0.5 | S | 0.38 | S | 1 |
| 75 | 4 | 1 | 0.25 | S | 0.25 | S | 0 |
| 76 | 13 | 1 | 0.19 | S | 0.25 | S | 1 |
| 77 | 42 | 1 | 0.19 | S | 0.25 | S | 0 |
| 78 | 10 | 1 | 0.38 | S | 0.38 | S | 0 |
| 79 | 13 | 1 | 0.5 | S | 0.25 | S | 0 |
| 80 | 42 | 1 | 0.25 | S | 0.5 | S | 0 |
| 81 | 42 | 1 | 0.38 | S | 0.38 | S | 0 |
| 82 | 104 | 1 | 256 | R | 1.5 | S | 3 |
| 83 | 153 | 1 | 0.25 | S | 0.38 | S | 2 |
| 84 | 4 | 1 | 256 | R | 1.5 | S | 3 |
| 85 | 6 | 1 | 0.125 | S | 0.38 | S | 0 |
| 86 | 42 | 1 | 0.5 | S | 0.38 | S | 0 |
| 87 | 37 | 4 | 256 | R | 1.5 | S | 3 |
| 88 | 6 | 1 | 0.125 | S | 0.25 | S | 2 |
| 89 | 13 | 1 | 0.125 | S | 0.125 | S | 2 |
| 90 | 239 | 1 | 0.125 | S | 0.5 | S | 0 |
| 91 | 1 | 2 | 0.38 | S | 0.047 | S | 2 |
| 92 | 4 | 1 | 0.19 | S | 0.25 | S | 0 |
| 93 | 4 | 1 | 0.125 | S | 0.38 | S | 0 |
| 94 | 13 | 1 | 0.125 | S | 0.25 | S | 0 |
| 95 | 239 | 1 | 0.25 | S | 0.25 | S | 2 |
| 96 | 6 | 1 | 1 | S | 0.25 | S | 2 |
| 97 | 13 | 1 | 1 | S | 0.19 | S | 2 |
| 98 | 1 | 2 | 1 | S | 2 | S | 1 |
| 99 | 4 | 1 | 0.38 | S | 0.032 | S | 0 |
| 100 | 6 | 1 | 0.5 | S | 0.25 | S | 2 |
| 101 | 4 | 1 | 256 | R | 1 | S | 3 |
| 102 | 103 | 1 | 0.125 | S | 0.25 | S | 2 |
| 103 | 421 | 1 | 256 | R | 1.5 | S | 3 |
| 104 | 1 | 2 | 0.25 | S | 0.38 | S | 1 |
| 105 | 2 | 1 | 0.19 | S | 0.25 | S | 0 |
| 106 | 55 | 1 | 0.047 | S | 0.25 | S | 1 |
| 107 | 37 | 4 | 0.19 | S | 1.5 | S | 0 |
| 108 | 4 | 1 | 256 | R | 2 | S | 3 |
| 109 | 104 | 1 | 256 | R | 1.5 | S | 3 |
| Isolate No. | **ST** | **Clade** | **Metronidazole**  **MIC- µg/ml** | **Metronidazole**  **S\R** | **Vancomycin**  **MIC- µg/ml** | **Vancomycin**  **S\R** | **Biofilm production capacity** |
| 110 | 3 | 1 | 0.019 | S | 0.25 | S | 2 |
| 111 | 104 | 1 | 256 | R | 1 | S | 3 |
| 112 | 4 | 1 | 256 | R | 1.5 | S | 3 |
| 113 | 6 | 1 | 256 | R | 1.5 | S | 2 |
| 114 | 4 | 1 | 256 | R | 256 | R | 3 |
| 115 | 13 | 1 | 0.125 | S | 0.38 | S | 0 |
| 116 | 1 | 2 | 0.5 | S | 0.38 | S | 2 |
| 117 | 54 | 1 | 0.094 | S | 4 | R | 2 |
| 118 | 59 | 1 | 1 | S | 3 | R | 3 |
| 119 | 2 | 1 | 0.047 | S | 0.5 | S | 0 |
| 120 | 60 | 1 | 0.064 | S | 4 | R | 3 |
| 121 | 43 | 1 | 0.5 | S | 8 | R | 3 |
| 122 | 39 | 4 | 0.5 | S | 4 | R | 3 |
| 123 | 43 | 1 | 1 | S | 3 | R | 3 |

**^*^** S=Susceptible; R= Reduced susceptibility**^.^**

^#^ ND- Not determined.

^&^ 0= non producer, 1=weak producer, 2= moderate producer, 3= strong producer.

**Table S2: Comparison of MIC between Etest and micro broth dilution**

|  | **Metronidazole**  **MIC (µg/mL)** | | | **Vancomycin**  **MIC (µg/mL)** | | |
| --- | --- | --- | --- | --- | --- | --- |
| **Isolate No.** | | **Etest** | **Micro broth dilution** | | **Etest** | **Micro broth dilution** |
| **5** | | 256 | >64 | | 0.75 | 0.5 |
| **6** | | 0.047 | 0.03 | | 0.25 | 0.25 |
| **8** | | 0.75 | 0.5 | | 1.5 | 1 |
| **25** | | 0.38 | 0.25 | | 0.75 | 0.5 |
| **37** | | 0.125 | 0.125 | | 0.38 | 0.25 |
| **38** | | 0.75 | 0.5 | | 0.016 | <0.03 |
| **39** | | 0.094 | 0.0625 | | 0.25 | 0.25 |
| **41** | | 256 | >64 | | 1 | 1 |
| **47** | | 256 | >64 | | 1.5 | 1 |
| **52** | | 256 | >64 | | 256 | >64 |
| **72** | | 0.094 | 0.0625 | | 0.19 | 0.125 |
| **118** | | 1 | 1 | | 3 | 2 |
| **120** | | 0.064 | 0.0625 | | 4 | 4 |
| **121** | | 0.5 | 0.5 | | 8 | 8 |
| **122** | | 0.5 | 0.5 | | 4 | 4 |
| **123** | | 1 | 1 | | 3 | 2 |

**Table S3: Expected and observed values for Chi square analysis**

| **Biofilm production capacity/ Metronidazole susceptibility** | **Expected values** | | **Observed values** | |
| --- | --- | --- | --- | --- |
|  | **S^*^** | **R** | **S** | **R** |
| **Non Producers** | 44.95 | 12.05 | 51 | 6 |
| **Weak Producers** | 10.25 | 2.75 | 12 | 1 |
| **Moderate Producers** | 18.93 | 5.07 | 22 | 2 |
| **Strong Producers** | 22.87 | 6.13 | 12 | 17 |

**^*^**S=Susceptible; R= Reduced susceptibility

**Table S4: Expected and observed values for Chi square analysis**

| **Biofilm production capacity/ Vancomycin susceptibility** | **Expected values** | | **Observed values** | |
| --- | --- | --- | --- | --- |
|  | **S^*^** | **R** | **S** | **R** |
| **Non Producers** | 51.90 | 5.10 | 57 | 0 |
| **Weak Producers** | 12.75 | 1.25 | 13 | 1 |
| **Moderate Producers** | 20.94 | 2.06 | 21 | 2 |
| **Strong Producers** | 26.41 | 2.59 | 21 | 8 |

**^*^**S=Susceptible; R= Reduced susceptibility

**Table S5: Comparison of antibiotic susceptibility pattern between different STs**

| **ST***  **(%)** | **Metronidazole,**  **n (%)**  **S^*^ R** | ***p* Value** | **Vancomycin,**  **n (%)**  **S R** | ***p* Value** |
| --- | --- | --- | --- | --- |
| **1** (5.9) | 5 (71.4) 2 (28.6) | **0.015**** | 7 (100) 0 | 0.391 |
| **2** (5) | 5 (83.3) 1 (16.7) |  | 6 (100) 0 |  |
| **4** (20.2) | 17 (70.8) 7 (29.2) |  | 23 (95.8) 1 (4.2) |  |
| **6** (6.7) | 6 (75) 2 (25) |  | 8 (100) 0 |  |
| **13** (8.4) | 10 (100) 0 |  | 10 (100) 0 |  |
| **37** (7.6) | 5 (55.5) 4 (44.5) |  | 8 (88.9) 1 (1.1) |  |
| **42** (8.4) | 9 (90) 1 (10) |  | 9 (90) 1 (10) |  |
| **104** (5.9) | 2 (40) 5 (60) |  | 7 (100) 0 |  |
| **Others^**^** (31.9) | 33 (86.8) 5 (13.2) |  | 30 (78.9) 8 (21.1) |  |

**^*^**S=Susceptible; R= Reduced susceptibility.

^**^ Others= ST3, ST8, ST10, ST12, ST17, ST35, ST43, ST54, ST55, ST59, ST60, ST103, ST153, ST239, ST421 and ST439.
